# Finesse in Gender Affirming Frontal Contouring With Corrugator Resection

**DOI:** 10.1093/asjof/ojae108

**Published:** 2024-11-11

**Authors:** Kevin G Hu, Jacqueline Ihnat, Neil Parikh, Melanie Vassallo, Mariana Almeida, David P Alper, Alexandre Prassinos, Ali Aral, Tito Vasquez, Michael Alperovich

## Abstract

Given the significance of forehead aesthetics to facial gender identification, frontal sinus setback and brow contouring are frequent components of facial feminization surgery (FFS) in transgender female patients. This study presents a safe and reliable technique for corrugator muscle resection during FFS to provide enhanced feminization of the forehead. This technique was performed in 12 patients between 27 and 58 years of age. Following bone reduction and sinus setback, the corrugator is located on the undersurface of the forehead flap and dissected through the reflected soft tissue of the forehead, with care taken to protect the supraorbital nerve. Patient satisfaction and complications, such as infection, poor wound healing, scarring, and desire for revision surgery, were assessed at follow-up visits. All patients showed improved feminization of the forehead. No complications were reported intraoperatively or postoperatively, and no patients required hospitalization beyond the expected overnight admission. No patients reported anesthesia or hypoesthesia in the distribution of the supraorbital nerve at 6 months postoperatively. Patients reported high satisfaction with their FFS outcomes. In conclusion, performing corrugator resection in conjunction with FFS is a safe and rapid technique that may significantly enhance the feminizing effects on the forehead of frontal sinus setback and brow contouring.

**Level of Evidence: 4 (Therapeutic):**

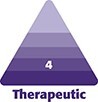

The fronto-orbito-nasal complex is the facial region most responsible for correct gender identification and consequently a main focus area in facial feminization surgery (FFS).^1-3^ Bony modifications typically include burring and frontal sinus setback to achieve forehead feminization.^4^ Addressing the corrugator supercilii muscle, responsible for brow furrowing, is a soft-tissue adjunct for further feminization, especially due to its greater likelihood of hypertrophy and increased mass in patients assigned male at birth.^5,6^ Corrugator resection in the context of FFS has been described in the literature only once before.^7^ In this article, the authors describe their technique for frontal contouring with corrugator resection as a component of FFS.

## METHODS

Corrugator supercilii resection is performed with frontal sinus setback and brow contouring/lifting. A traditional trichophytic incision is made descending to the subperiosteal plane, with further dissection progressing caudally to the supraorbital rim. The supraorbital bundle is identified and protected. An osteotomy may be performed to free the supraorbital bundle in patients with a supraorbital foramen; in patients with a notch, the neurovascular bundle is reflected downwards. Frontal sinus setback and brow contouring are performed in the typical fashion prior to corrugator resection (Video).

Corrugator muscle position is estimated relative to the medial brow superior to the orbital rim. Blunt dissection of the subgaleal layer and targeted electrocautery reveal the deep surface of the corrugator muscle with oblique muscle fibers. Supraorbital bundle branches are identified and reflected away as they course lateral to the dissection site. The muscle is isolated and resected with electrocautery. Continued resection of the corrugator laterally further reduces brow volume and decreases the likelihood of recurrent corrugator hypertrophy. Further brow flattening can be achieved by resecting the depressor supercilii superficial and medial to the corrugator.

Once resection is completed, brow lift and hairline reduction are performed, followed by skin closure in the traditional fashion. A 10-flat JP drain is placed in the subgaleal scalp. Patients are observed in the hospital overnight and the drain removed before discharge.

This retrospective study was exempt from IRB review due to its noninterventional nature. It adhered to the principles of the Declaration of Helsinki, and written informed consent was obtained from all patients for their procedures and use of data and images for research and publication.

## RESULTS

Twelve patients aged 27 to 58 years underwent FFS with corrugator resection at a single institution by a single surgeon (Table 1). All patients had at least 1 year of hormone replacement therapy and lived as their experienced gender prior to surgery. No patients received botulinum toxin treatments before FFS. Corrugator resection added <10 min of operative time in all patients.

**Table 1.** Patient Characteristics and Procedures Received

Patient number	1	2	3	4	5	6	7	8	9	10	11	12
Age	41	32	36	29	27	49	36	33	40	21	46	58
>1 year HRT	✓	✓	✓	✓	✓	✓	✓	✓	✓	✓	✓	✓
>1 year as gender	✓	✓	✓	✓	✓	✓	✓	✓	✓	✓	✓	✓
Past GAS												
Top surgery			✓				✓	✓				✓
Bottom surgery	✓								✓			✓
Procedures received												
Frontal sinus setback	✓	✓	✓	✓	✓	✓	✓	✓	✓	✓	✓	
Corrugator resection	✓	✓	✓	✓	✓	✓	✓	✓	✓	✓	✓	✓
Brow lift	✓	✓	✓	✓	✓	✓	✓	✓	✓	✓	✓	✓
Brow contouring	✓	✓	✓	✓	✓	✓	✓	✓	✓	✓	✓	
Hairline reduction	✓	✓	✓	✓	✓	✓	✓	✓	✓	✓	✓	✓
Rhinoplasty	✓	✓	✓	✓	✓	✓	✓	✓	✓		✓	
Lip lift	✓			✓		✓						
Genioplasty	✓	✓		✓	✓	✓	✓	✓	✓	✓	✓	
Chondrolaryngoplasty		✓	✓		✓	✓	✓					
Gonial angle reduction	✓		✓	✓	✓		✓					

GAS, gender affirmation surgery; HRT, hormone replacement therapy.

**Figure 1.**
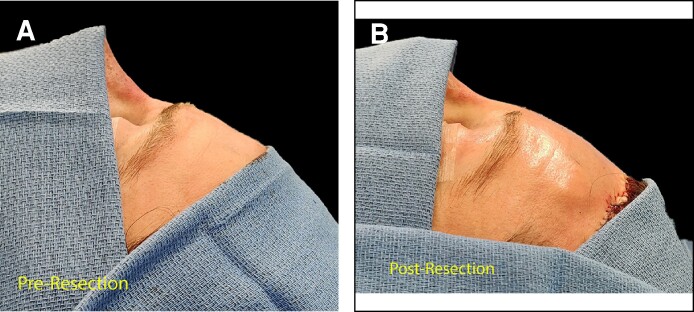
The comparison of profile views of the forehead (A) before and (B) after facial feminization surgery. The forehead after corrugator resection has a smoothly convex profile, in contrast to the discrete step-off present at the brow in the before picture.

After surgery, the forehead is more uniformly convex, particularly at the brow (Figure 1), and all patients reported satisfaction with forehead appearance postoperatively. The average follow-up time was 8.8 ± 2.0 months, and no patients reported complications, infections, asymmetry, or loss of sensation of the forehead or brow.

## DISCUSSION

Upper-third FFS reduces masculine features through bony modifications, including frontal sinus setback, forehead burring, and supraorbital burring,^4^ and soft-tissue modification, such as hairline advancement and brow lifts.^8^ Corrugator resection also feminizes the forehead and brow in transgender females. These results show that corrugator resection is a safe adjunct to FFS, as the risk presented to the supraorbital nerve and the orbit is similar to other upper-third bony contouring procedures. The increased access available through a coronal or trichophytic incision, compared with the more common transpalpebral approach for facial rejuvenation, further enhances this technique's safety profile.^9,10^

**Figure 2.**
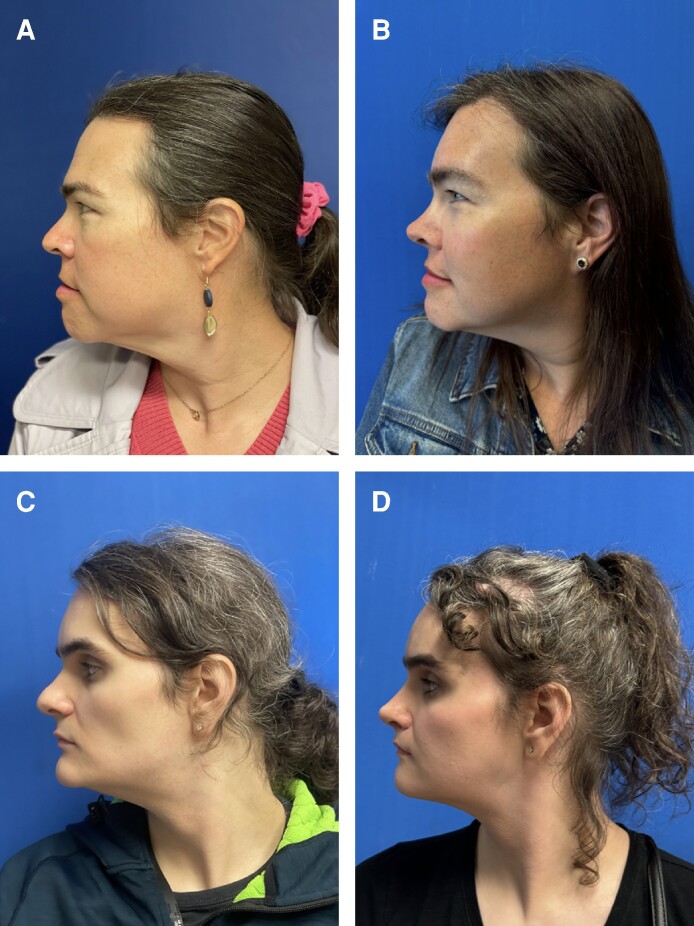
The profile view of the forehead at a 6-month follow-up visit for 2 transgender female patients, aged 41 and 29. Note the increased continuous convexity of the forehead and decreased brow furrowing after surgery (B, D) compared with before surgery (A, C).

Corrugator resection reduces glabellar prominence and decreases supraorbital concavity, creating a more feminine convex forehead (Figure 2). Corrugator resection can decrease glabellar volume by about 3 mL, with fat grafting as an option to smooth out irregularities in glabellar shape.^9^ Resection also elevates and feminizes the brow by diminishing brow furrowing and depression. Specifically, while masculine brows are horizontal and at the supraorbital ridge, feminine brows typically begin medially at or inferior to the supraorbital ridge and ascend laterally with a peak between the middle and lateral thirds.^8,11^

Corrugator resection also decreases forehead rhytids, simultaneously feminizing and de-aging the forehead.^12,13^ More prominent horizontal rhytids of the forehead in males have been attributed to increased frontalis activation to compensate for corrugator hypertrophy and a lower brow.^14,15^ Diminishing excessive frontalis activation reduces rhytids, a particularly desirable effect in older patients. Although chemodenervation with botulinum toxin in the corrugator achieves the same effect, it is temporary and costly.^13^ Corrugator resection can thus be a more economical treatment for patients who would otherwise seek frequent chemodenervation.

**Figure 3.**
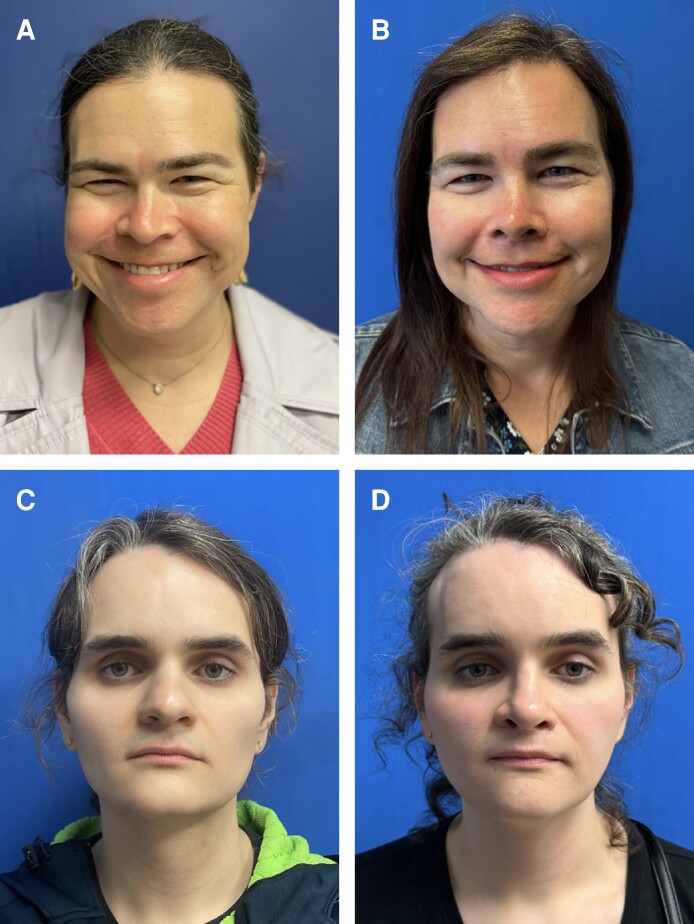
Frontal view of the forehead at a 6-month follow-up visit for 2 transgender female patients, aged 41 and 29. Note the reduction in glabellar lines without the manifestation of a “surprised look” due to excessive frontalis activation after surgery (B, D) compared with before surgery (A, C).

Corrugator resection achieves optimal outcomes in concert with several different procedures, including brow lift and hairline reduction to achieve facial harmony and aesthetic improvement (Table 1). Corrugator resection amplifies these effects, especially at the medial brow. Overresection may lead to excessive brow elevation and a “surprised” look, although no patients in this series expressed this concern (Figure 3).^16^ Thus, discussing the patient's aesthetic goals given their preoperative facial features is critical.

Corrugator resection also represents an opportunity for migraine treatment in a unique patient population. Transgender females on hormone replacement therapy experience migraines at the elevated level of cisgender females,^17^ likely through changes in neuroexcitability and pain perception.^18^ Concurrent removal of the supratrochlear and supraorbital arteries can significantly improve migraine symptoms.^9^

This work is limited by a relatively short follow-up period, a small patient sample, concurrent frontal sinus setback, and the use of nonstandardized measures of patient satisfaction. Future studies should focus on quantifying the change attributable to corrugator resection alone.

This technique is rapid, adding ∼10 min of operative time, and convenient, requiring no additional incisions and minimal additional dissection. We now offer corrugator resection routinely as part of our complement of upper-third gender-affirming procedures.

## CONCLUSIONS

Corrugator resection is an adjunct to FFS that may offer enhanced forehead feminization in conjunction with classical techniques such as frontal sinus setback and brow contouring. By taking advantage of an existing hairline trichophytic incision, this procedure is both safe and rapid, yielding improved forehead aesthetics without increased cost or risk to the patient.
